# Fabrication of malleable three-dimensional-printed customized bolus using three-dimensional scanner

**DOI:** 10.1371/journal.pone.0177562

**Published:** 2017-05-11

**Authors:** Jae Won Park, Se An Oh, Ji Woon Yea, Min Kyu Kang

**Affiliations:** 1 Department of Radiation Oncology, Yeungnam University College of Medicine, 170 Hyeonchung-ro, Nam-gu, Daegu, South Korea; 2 Department of Radiation Oncology, Kyungpook National University School of Medicine, 807 Hoguk-ro, Buk-gu, Daegu, South Korea; George Washington University, UNITED STATES

## Abstract

A three-dimensional (3D)-printed customized bolus (3D bolus) can be used for radiotherapy application to irregular surfaces. However, bolus fabrication based on computed tomography (CT) scans is complicated and also delivers unwanted irradiation. Consequently, we fabricated a bolus using a 3D scanner and evaluated its efficacy. The head of an Alderson Rando phantom was scanned with a 3D scanner. The 3D surface data were exported and reconstructed with Geomagic Design X software. A 3D bolus of 5-mm thickness designed to fit onto the nose was printed with the use of rubber-like printing material, and a radiotherapy plan was developed. We successfully fabricated the customized 3D bolus, and further, a CT simulation indicated an acceptable fit of the 3D bolus to the nose. There was no air gap between the bolus and the phantom surface. The percent depth dose (PDD) curve of the phantom with the 3D bolus showed an enhanced surface dose when compared with that of the phantom without the bolus. The PDD of the 3D bolus was comparable with that of a commercial superflab bolus. The radiotherapy plan considering the 3D bolus showed improved target coverage when compared with that without the bolus. Thus, we successfully fabricated a customized 3D bolus for an irregular surface using a 3D scanner instead of a CT scanner.

## Introduction

The skin-sparing effect of megavoltage-photon beams in radiotherapy forms a major benefit in the treatment of deep-seated tumours; however, skin-sparing reduces the target coverage of superficial tumours [1]. Consequently, a bolus is widely used to enhance the target coverage for superficial targets. A commercial flat bolus cannot easily be applied on irregular surfaces, where unwanted air gaps exist between the bolus and body surface, thereby resulting in decreased surface doses [2]. In order to solve this issue, three-dimensional boluses (3D boluses) have been successfully fabricated and applied to Rando phantoms with the use of printing technologies [3]. In this regard, the application of 3D printing technology to radiation oncology has formed a strong research focus [4–9].

Recently, our clinical experience of a 3D bolus applied to intensity-modulated radiotherapy for a patient with Kimura’s disease revealed certain problems regarding the fabrication of the 3D bolus [10]: (1) The design of a 3D bolus with the use of computed tomography (CT) scans leads to unwanted radiation exposure of the patient. (2) The process from CT scanning to the generation of a STereoLithography (STL) file for 3D printing also requires heavy computation. (3) Further, the use of hard printing materials such as acrylonitrile butadiene styrene (ABS) can cause discomfort to the patient. (4) Moreover, it is difficult to minimize the air gaps between the body surface and the bolus. In this context, here, we explore the radiotherapy application possibility of a 3D bolus made of malleable material with the aid of a 3D scanner.

## Materials and methods

### Scanning and printing procedure

The head of an Alderson Rando phantom (The Phantom Laboratory, Salem, NY, USA) was utilized for the fabrication process. Because the phantom is dark in colour, powder spray was applied to obtain a better resolution and 3D image accuracy. Next, the optical image data of the phantom were acquired with the use of an Artec Spider 3D scanner (Artec Group, Luxembourg) and exported to the workstation. The surface image data were reconstructed, and a customized 3D bolus was designed with the Geomagic Design X version 2014 software package (Geomagic Inc., Morrisville, NC, USA). A 5-mm-thick customized bolus of the nose was designed by isotropic expansion from the surface.

**Fig 1:**
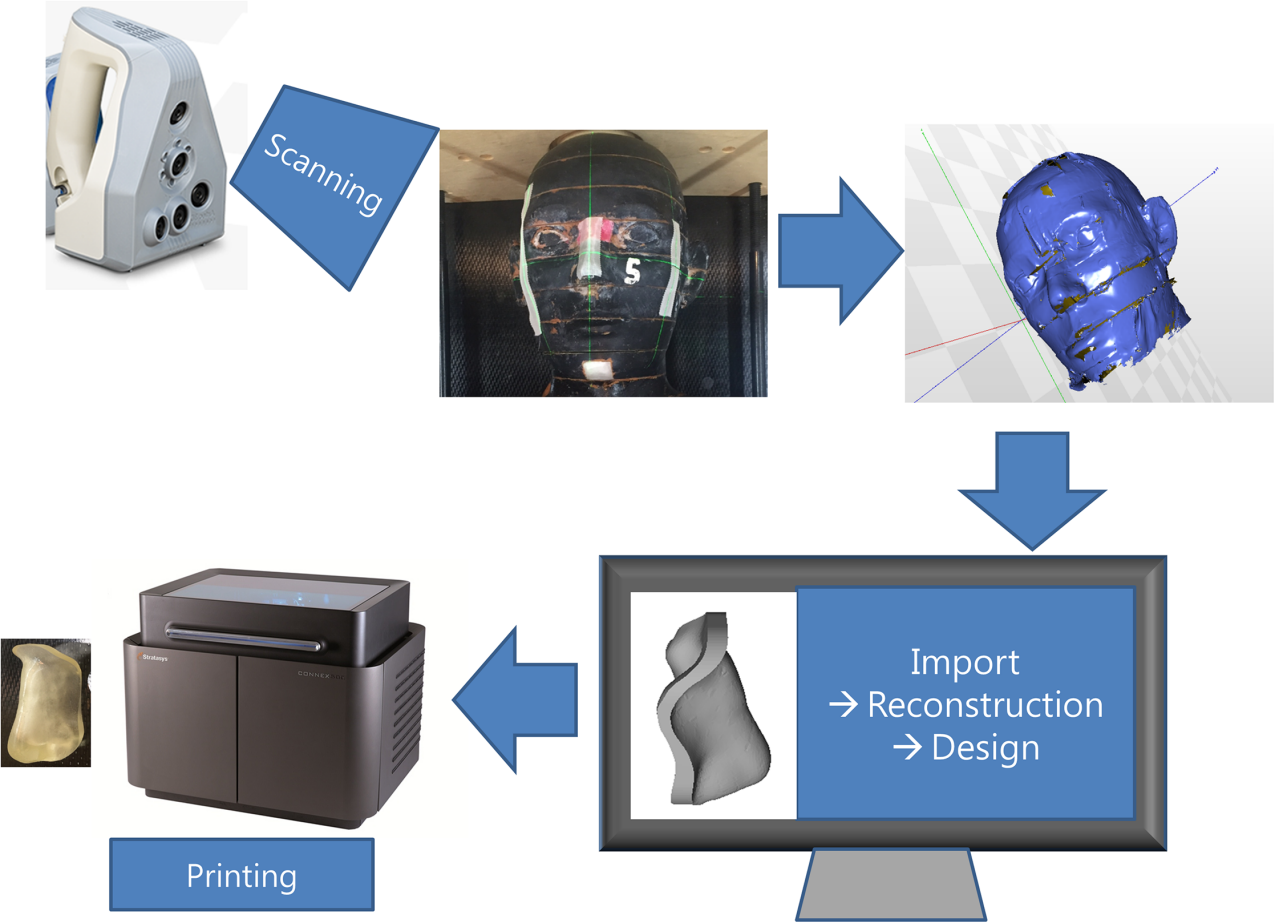
Fabrication process of 3D bolus using 3D scanner. 3D bolus, three-dimensional-printed customized bolus.

The designed bolus was printed with the use of a Stratasys Objet500 Connex3 with the use of PolyJet technology (Stratasys Ltd., Eden Prairie, MN, USA) with the malleable ‘rubber-like’ printing material, Tango Plus (Stratasys Ltd.), to reduce patient discomfort and unwanted air gaps. Fig 1 shows the overall processes involved in the bolus fabrication.

### Dosimetric evaluation

CT simulations of the Rando phantom with and without the 3D bolus were performed to evaluate the dosimetric properties of the 3D bolus. The nose was delineated as the target. Radiotherapy plans with anterior one portal were generated in the Eclipse radiotherapy treatment planning system version 8.6 (Varian Medical System, Palo Alto, CA, USA) with the use of the anisotropic analytical algorithm. The prescription dose was 200 cGy, and it was normalized for 95% of the prescribed dose to cover 100% of the target volume in the plan with the 3D bolus. The plan without the bolus was normalized to make its maximal relative dose the same as that of the plan with the 3D bolus. The following dosimetric parameters were estimated for both cases: maximal dose (D_max_), mean dose (D_mean_), minimum dose (D_min_), V_95%_ (volume receiving at least 95% of the prescription dose), and V_90%_.

To evaluate the percent depth dose (PDD) profiles with the use of the 3D bolus, we analysed the PDD profiles under the three following conditions in the Rando phantom: without a bolus, with a commercial superflab bolus, and the 3D bolus. Gafchromic EBT2 films (ISP Corporation, Wayne, NJ, USA) were cut and inserted along the vertical direction in the phantom. After irradiation, the films were scanned in the RGB mode with an Epson 10000XL flatbed scanner according to the manufacturer recommendations for the film. The scanned images were analysed with the use of the Omnipro I’mRT Version 1.7 (IBA Dosimetry, Schwarzenbruck, Germany) package. A more detailed description of the film dosimetry with the use of the Gafchromic EBT2 films is provided in a previous study [11].

## Results

### 3D-printed customized bolus (3D bolus) using 3D scanner

**Fig 2:**
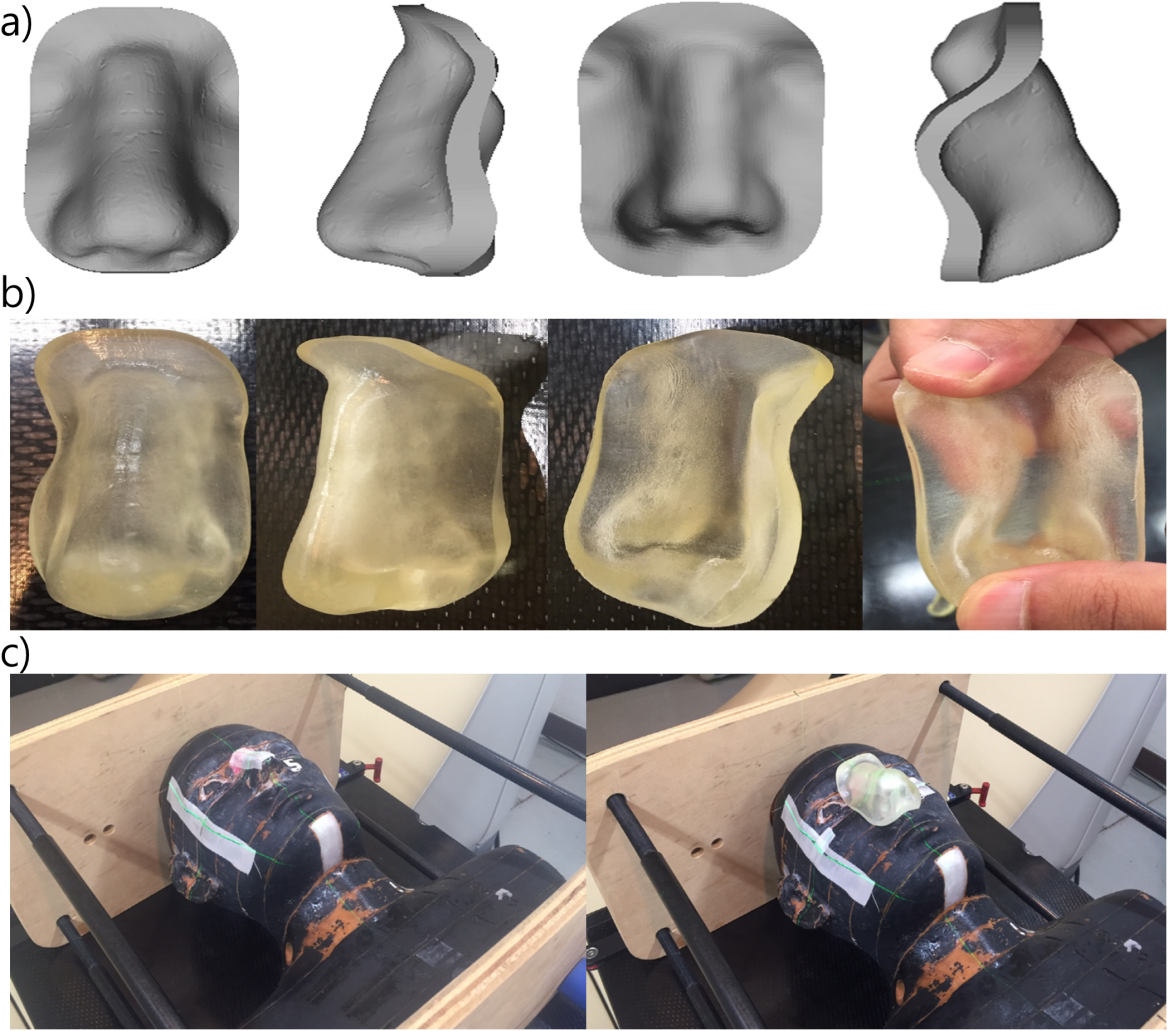
3D bolus fabricated with 3D scanner. (A) STL file view of the designed 3D bolus, (B) printed result obtained with malleable material, Tango Plus, and (C) CT simulation scan setup of the bolus on the Rando phantom. 3D bolus, three-dimensional-printed customized bolus; STL, STereoLithography; CT, computed tomography.

**Fig 3:**
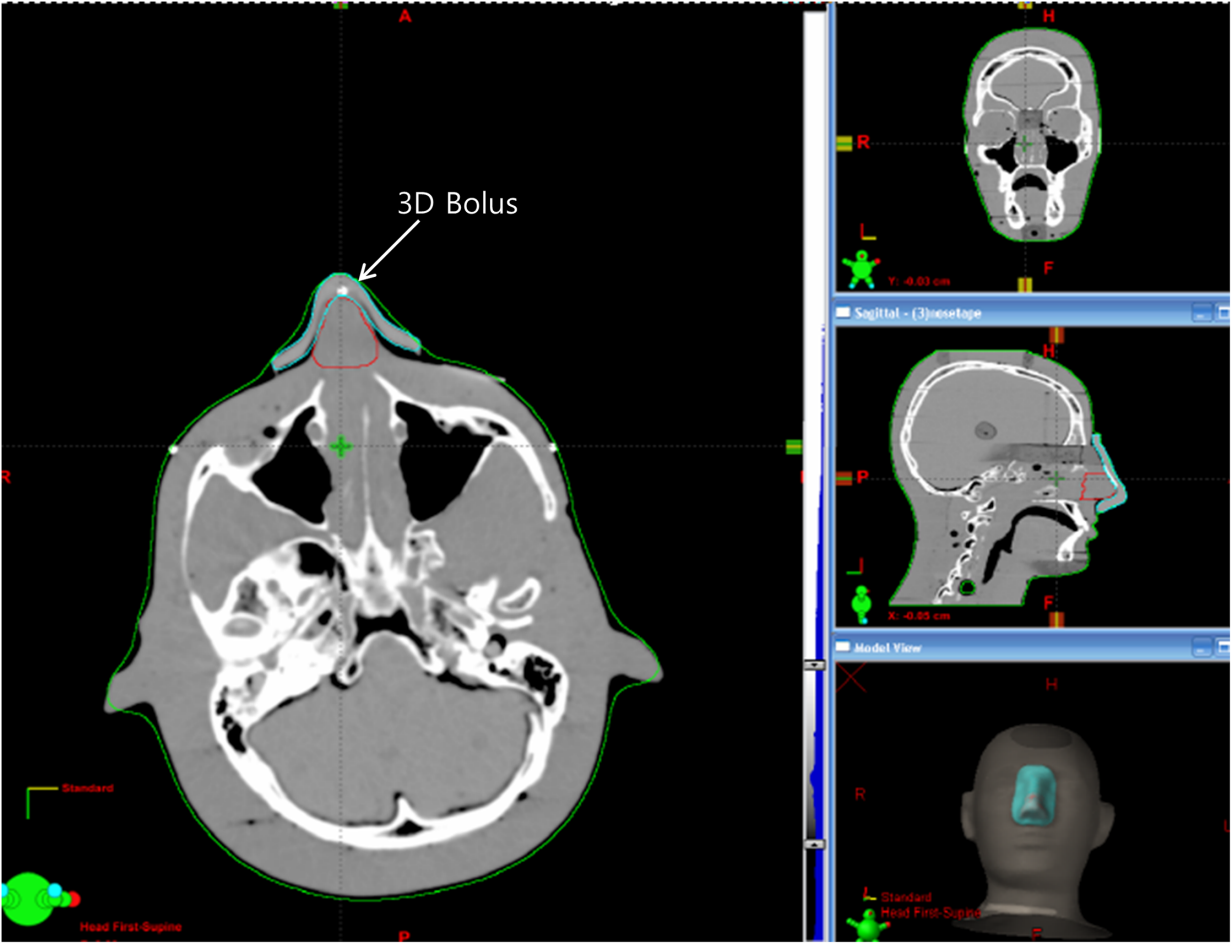
CT simulation image. The 3D bolus suitably fits the irregular surface. CT, computed tomography; 3D bolus, three-dimensional-printed customized bolus.

Fig 2A and 2B show the design of the 3D bolus and the corresponding printed result. The Rando phantom set up for CT simulation is shown in Fig 2C. The 3D bolus was malleable, and it suitably fitted onto the nose of the Rando phantom without any air gaps (Fig 3).

### Plan and dosimetric results

**Fig 4:**
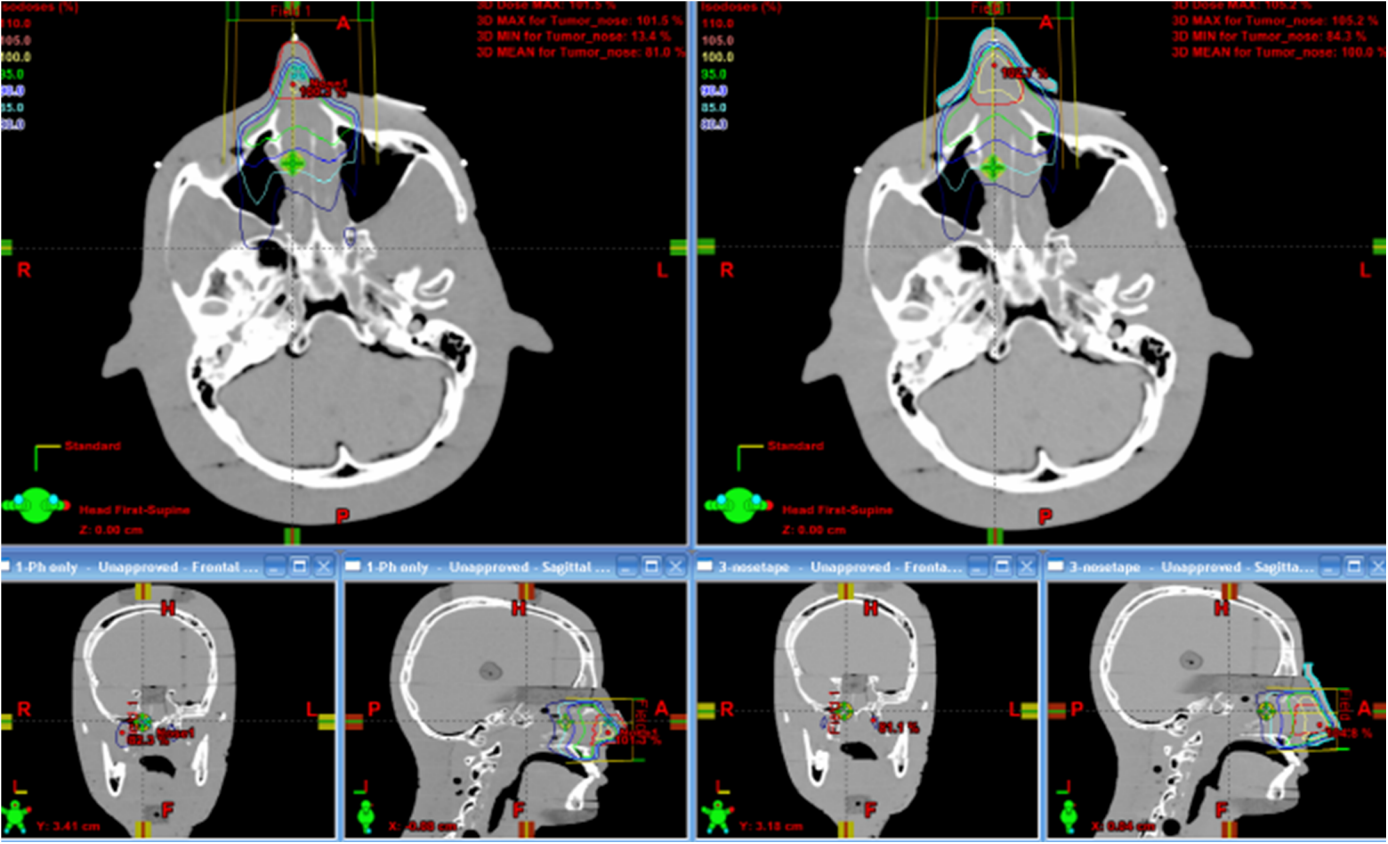
Isodose lines corresponding to the two radiation therapy plans with and without the 3D bolus. The 3D bolus sufficiently enhanced the surface dose, resulting in superior target coverage (Isodose lines: pink, 105%; yellow, 100%; green, 95%; blue, 90%; sky-blue, 80%; orange, 70%; white, 50%). 3D bolus, three-dimensional-printed customized bolus.

**Fig 5:**
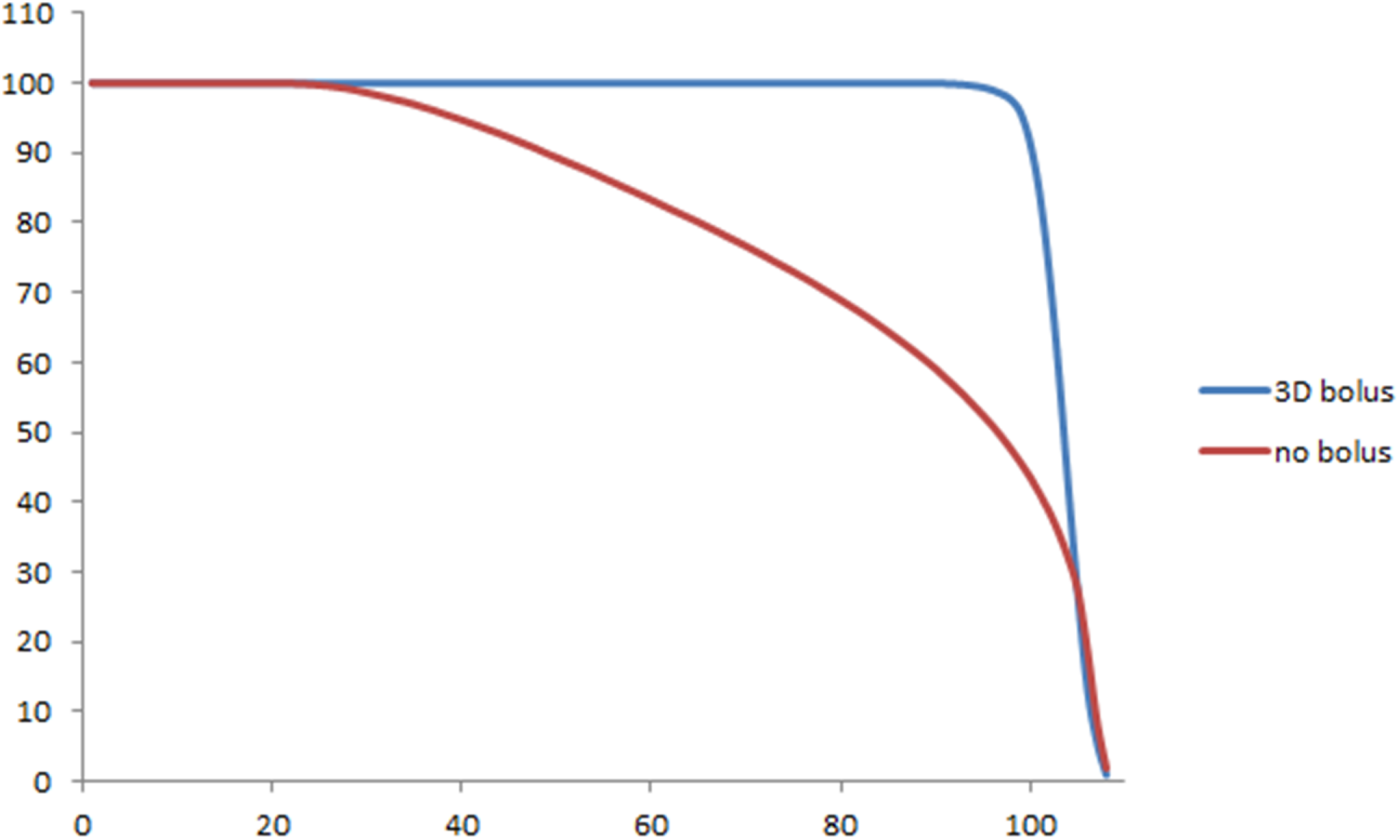
DVH of the target corresponding to the plans with and without the 3D-printed bolus. DVH, dose–volume histogram; 3D bolus, three-dimensional-printed customized bolus.

Fig 4 shows the isodose lines corresponding to the plans with and without the bolus. We observe that the target coverage is better with the 3D bolus. Fig 5 shows the dose–volume histogram of the nose with and without the 3D bolus, and Table 1 summarizes the relevant dosimetric parameters for both plans.

**Table 1:** Dosimetric parameters of the two plans: without bolus and with the 3D bolus.

Parameter	No bolus	3D bolus
D_max_ (%)	107.7	107.7
D_mean_ (%)	85.7	102.3
D_min_ (%)	14.2	86.3
V_95%_(%)	50.8	86.3
V_90%_(%)	57.8	99.0

3D, three-dimensional.

**Fig 6:**
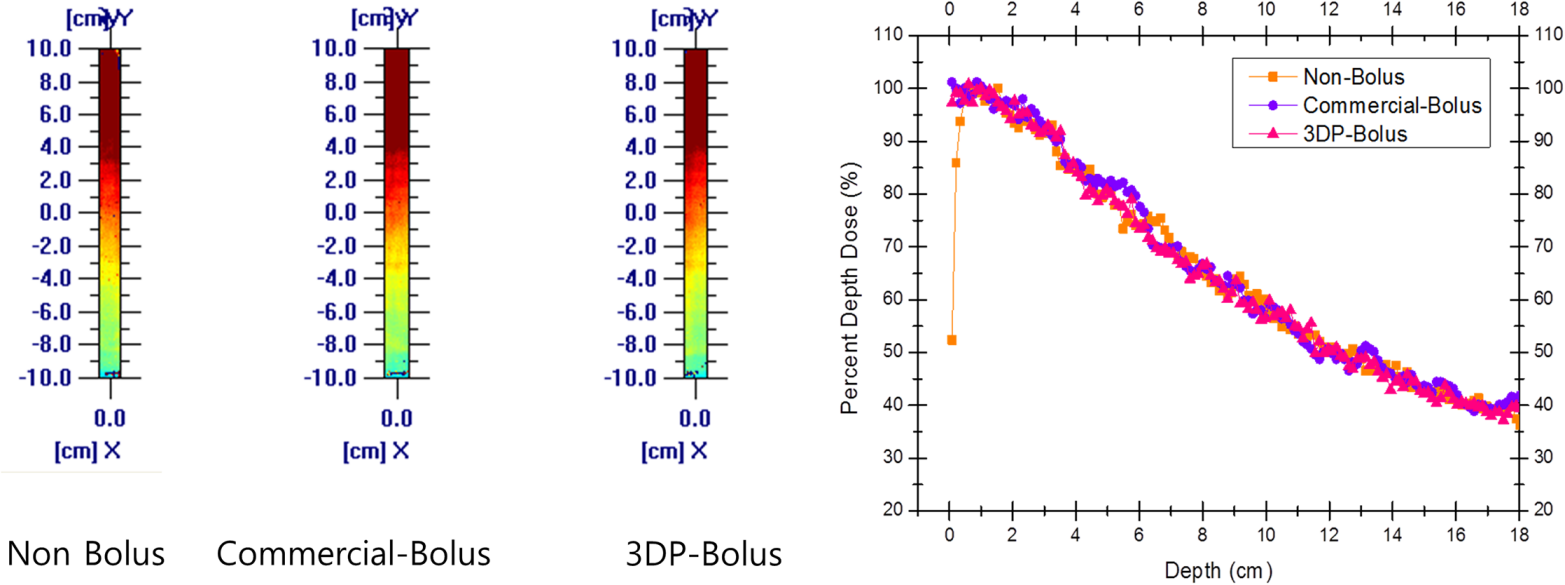
Percent depth dose (PDD) analysis with the use of Gafchromic EBT film. The 3D-printed bolus sufficiently enhanced the surface dose, and its PDD was comparable with that of a commercial flat bolus. The PDD without a bolus shows significant under-dosing to the surface area. PDD, percent depth dose; 3D, three-dimensional.

From the PDD analysis, we observed that the 3D bolus successfully enhanced the surface dose. Further, the PDD of the 3D bolus was comparable with that of the commercial bolus (Fig 6). Moreover, the PDD profiles corresponding to the measured dose in the EBT film and the calculated dose in treatment planning system were similar (S1 Fig).

## Discussion

The build-up effect is considered a major benefit of megavolt photon beams because of reduction in the skin dose, thus resulting in reduced skin toxicity. However, this effect can jeopardize target coverage of superficial tumours, and therefore, a commercial bolus is commonly used in such situations to increase the surface dose in radiation oncology clinics [12]. However, difficulties in fitting a commercial bolus can result in unwanted air gap formation over irregular surfaces, which can be detrimental to radiotherapy planning; a previous study reported that a 10-mm air gap was resulted in a 10% reduction in the surface dose [2].

This study examines the possibility of using a 3D scanner for the fabrication of a customized bolus. In this regard, Kim et al. used CT scans for the fabrication of a 3D bolus for the irregular surface of a Rando phantom [3]. We have previously reported our fabrication experience of a 3D bolus using CT scans [10]. In comparison with the process based on the CT scan, the fabrication process based on a 3D scanner is simple and less time-consuming because this approach can skip several steps in the normal process from the contouring of the body surface to the generation of the STL file. In addition, a benefit of using a 3D scanner is that there is no additional radiation exposure. Although radiation exposure due to CT is a small fraction of the amount by radiation therapy, efforts to reduce unnecessary radiation exposure are needed.

In this study, we used PolyJet printing technology to print a bolus made of a malleable printing material, Tango Plus. Kim et al. have previously used PolyJet technology to print a malleable heart model [13]. In our previous clinical study, the use of hard materials such as ABS*plus* (Stratasys Ltd.) for fabrication of the 3D bolus in a clinical case resulted in the patient experiencing pain, and further, the air gap between the body surface and the bolus could not be suitably minimized [10]. Consequently, we realised that a more malleable material may be needed to closely adapt the bolus to the surface of the target region.

Our dosimetric analysis in this study showed that satisfactory target coverage could be achieved with the proposed 3D bolus. Moreover, our PDD analysis with EBT film confirmed that the bolus fabricated from Tango Plus could increase the surface dose effectively, and this dose was comparable with that of a commercial bolus. In this context, Kim et al. have reported that a 3D-printed customized bolus provides a better fit for the nose, leading to improvements in D_min_, D_mean_, D_90%_, and V_90%_ [3].

In conclusion, we successfully fabricated a 3D bolus to cover an irregular surface using a 3D scanner instead of CT scans. The fabrication process was simple and fast. The bolus, made of the malleable material Tango Plus, suitably fitted the surface, and the surface dose was sufficiently enhanced. Thus, we believe that the use of a 3D scanner and malleable materials can be seriously considered for the fabrication of customized boluses.

## Supporting information

S1 Fig Percent depth dose (PDD) profile analysis on central axis. Calculated dose in treatment planning system (above) and measured dose in EBT film (below). 3DP bolus, three-dimensional-printed customized bolus.
(TIF)
