# Supplementary figures and images for: Fabrication of malleable three-dimensional-printed customized bolus using three-dimensional scanner
Source: PLoS One. 2017 May 11;12(5):e0177562. doi: 10.1371/journal.pone.0177562 (PMC5426771; doi:10.1371/journal.pone.0177562)

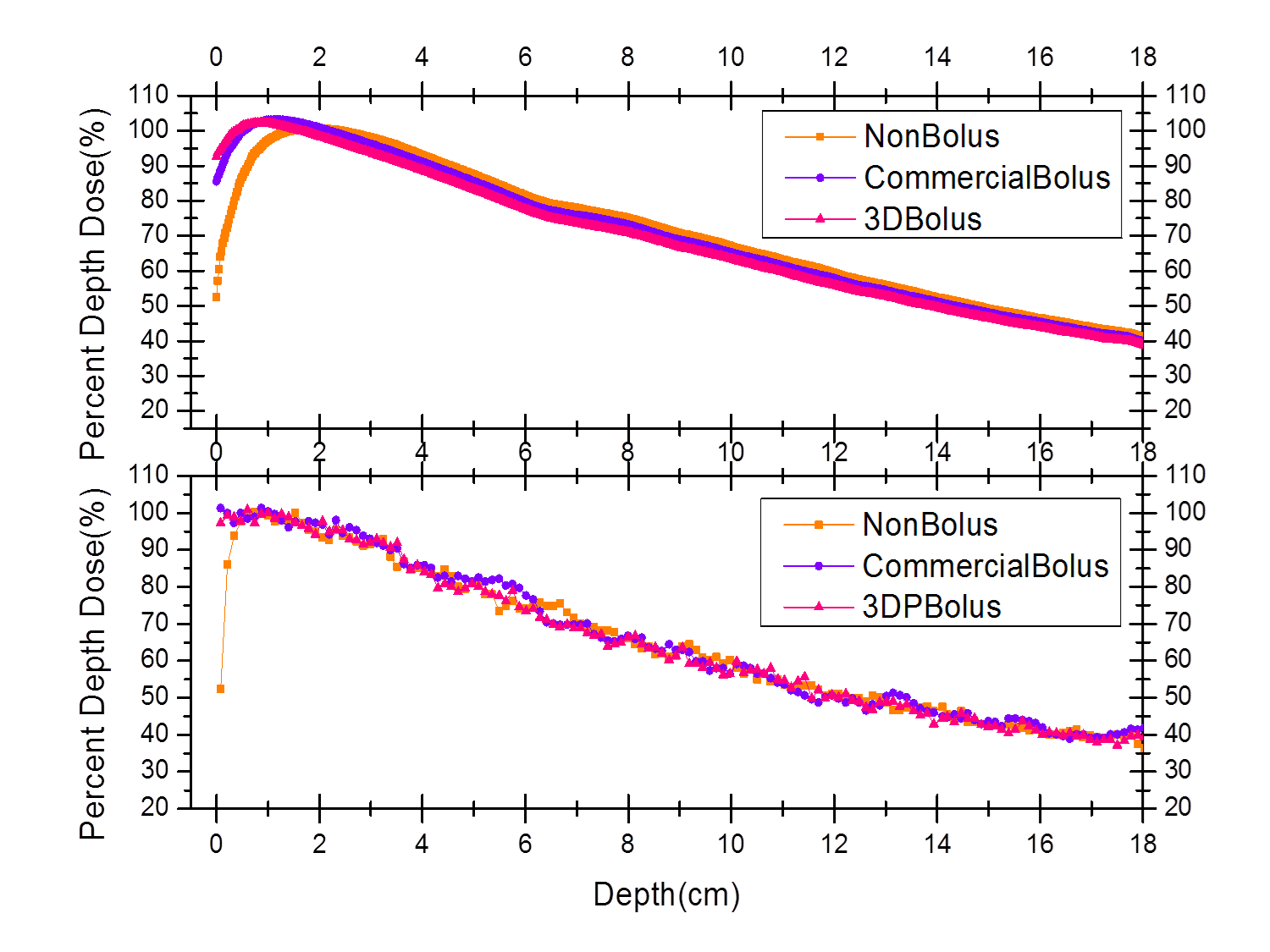

Supplement: S1 Fig — Calculated dose in treatment planning system (above) and measured dose in EBT film (below). 3DP bolus, three-dimensional-printed customized bolus. (TIF) [file pone.0177562.s001.tif]
